# Gum Mezquite as a Substitute for Gum Arabic

**Published:** 1854-12

**Authors:** George G. Shumard


### ART. IV.—GUM MEZQUITE AS A SUBSTITUTE FOR GUM ARABIC.

BY GEORGE G. SHUMARD, M. D.

FORT SMITH, ARK., Oct. 20, 1854.

PROF. YANDELL:—

Dear Sir, having just returned from an exploration of the head waters of the Big Witchita and Brazos rivers, I hasten to communicate to you a discovery which may hereafter prove to be of considerable importance, and which I would be pleased to have you announce in the *Western Journal of Medicine and Surgery*..

While on our journey I was surprised to find exuding from the *Mezquite Tree*, (a well known tree of the plains,) a light amber-colored gum, which upon examination I found to possess properties very similar to, if not identical with, those of the best gum Acacia. Delighted with the discovery, I resolved to follow up the investigation, and have succeeded in collecting a mass of facts, a portion of which I may hereafter embody in a separate article for your journal. For the present I will merely refer you to the inclosed copy of a note to Ex-Gov. DREW, Superintendent of Indian Affairs.

Along with this I send you a fair average sample of the gum which I would be pleased to have properly tested either by yourself or Prof. SILLIMAN. The different lumps in the sample are all from the same tree. I have employed it medicinally in several instances and find it answers the purpose of the best gum-acacia. With water it forms a beautiful mucilage which for medicinal purposes I have even thought superior to that of gum-acacia, while for a proof of its great tenacity I will refer you to this package, with which it is sealed.

My scientific collection consisting of speciment in zoology, conchology, geology, &c., (besides being very large) is highly interinteresting. The fossils alone number several thousand specimens and will no doubt furnish a number of new species; they are mostly from the cretaceous and carboniferous formations. In distributing them to my friends I will not forget you.

Respectfully yours,
GEO. G. SHUMARD.

---

FORT SMITH, Oct. 18, 1854.

SIR:—I cheerfully comply with your request to furnish for the use of the Indian Department a short description of the *Gum Mezquite* discovered during our recent expedition to the head waters of the Big Witchita and Brazos rivers.

This gum, (for which I propose the name of *Gum Mezquite*), is believed to occur in inexhaustible quantities, and will no doubt hereafter prove a valuable source of revenue to the State of Texas, New Mexico and the adjacent Indian territory, besides affording employment to the different tribes of indians now roving upon the plains, many of whom would no doubt be glad to gather and deliver it to the different frontier posts for a very small compensation.

The *Mezquite Tree*, from which the gum is obtained, is by far the most abundant tree of the plains, covering thousands of miles of surface, and always flourishes most luxuriantly in elevated and dry regions. The gum exudes spontaneously in a semi-fluid state from the bark of the trunk and branches, and soon hardens by exposure to the atmosphere, forming more or less rounded and variously colored masses, weighing each from a few grains to several ounces. These soon bleach and whiten upon exposure to the light of the sun, finally becoming nearly colorless, semi-transparent, and often filled with minute fissures. Specimens collected from the trunks of the trees were generally found to be less pure and more highly colored than when obtained from the branches. The gum may be collected during the months of July, August and September, but the most favorable period for that purpose is in the latter part of August, when it may be obtained in the greatest abundance and with but little trouble. The quantity yielded by each tree varies from an ounce to three pounds, but incisions made in the bark not only greatly facilitate its exudations but cause the tree to yield a much greater amount. As it is, a good collector would probably be able to gather from ten to twenty pounds in a day, were incisions resorted to probably double the amount might be obtained.

I have thus presented a few of the leading facts connected with this interesting discovery; should you desire still farther information upon the subject it will give me much pleasure to furnish it.

I am with much respect,

Your obedient servant,

GEO. G. SHUMARD.

HON. THOMAS DREW,

Superintendant of Indian Affairs.

